# Supplementary material for: Understanding the Implementation of “Sit Less at Work” Interventions in Three Organisations: A Mixed Methods Process Evaluation
Source: Int J Environ Res Public Health. 2021 Jul 9;18(14):7361. doi: 10.3390/ijerph18147361 (PMC8304152; doi:10.3390/ijerph18147361)
Supplement: Supplementary file 1 [file ijerph-18-07361-s001.zip › Eval Paper_Table S2.pdf]

Table S2a. Charity 12-week “Sit Less at Work” intervention summary

Actions to be initiated as appropriate at the start or throughout the 12-weeks:

- *At the start: communications sent to managers and to all staff informing of the sit less initiative (Org)*
- *Hold any team meetings during this time stood-up or incorporate standing/moving into meeting agendas, or conduct standing/walking 1:1s (Org)*
- *Changes in policies and guidelines (working document) (Org)*

Week	Action 1	Action 2	Action 3	Action 4
**1**	Email from Chief Exec to encourage and support staff to take short, regular breaks<br>(Intra & Org)			
**2**	Email from Chief Exec to encourage and support staff to take short, regular breaks	Personal targets for steps/day and track activity using FitBit/pedometers and record steps each day<br>(Intra & Inter)		
**3**	Email from Chief Exec to encourage and support staff to take short, regular breaks	Celebrate changes/achievements – tell us your sit less stories<br>(Inter & Org)		
**4**	Email from Chief Exec to encourage and support staff to take short, regular breaks	Personal targets for steps/day and track activity using FitBit/pedometers and record steps each day	Lunchtime walking or running group<br>(Inter)	

**5**	Celebrate changes/ achievements – "tell us your sit less stories"	Lunchtime walking or running group		
**6**	Email from Chief Exec to encourage and support staff to take short, regular breaks	Personal targets for steps/day and track activity using FitBit/pedometers and record steps each day	Lunchtime walking or running group	Clear office/desk policy (Intra, Org & Env)
**7**	Celebrate changes/ achievements – "tell us your sit less stories"	Lunchtime walking or running group	Clear office/desk policy	
**8**	Email from Chief Exec to encourage and support staff to take short, regular breaks	Personal targets for steps/day and track activity using FitBit/pedometers and record steps each day	Lunchtime walking or running group	Clear office/desk policy
**9**	Celebrate changes/ achievements – "tell us your sit less stories"	Lunchtime walking or running group	Clear office/desk policy	
**10**	Email from Chief Exec to encourage and support staff to take short, regular breaks	Personal targets for steps/day and track activity using FitBit/pedometers and record steps each day	Lunchtime walking or running group	Clear office/desk policy
**11**	Celebrate changes/ achievements – "tell us your sit less stories"	Lunchtime walking or running group	Clear office/desk policy	
**12**	Email from Chief Exec to encourage and support staff to take short, regular breaks	Personal targets for steps/day and track activity using FitBit/pedometers and record steps each day	Lunchtime walking or running group	Clear office/desk policy

Table S2b. Charity detailed action plan

Action	Content	Timing	Management involvement	Barriers	How to overcome barriers	Who to action
**Take regular short breaks (Intra & Org)**	Incorporate various other suggestions here into an email from Chief Exec to encourage staff to take regular short breaks e.g. use stairs not the lift, make your own drinks, drink more water, "walk, talk, email", lunch away from your desk / lunchtime walk, walking/standing meetings	Day 1, week 1 and weekly reminder emails after that	Chief Exec to send email	Making own drinks as there is a culture of rounds of drinks – will require agreement among teams to do this, have to make sure everyone is aware.	Could be raised at team meeting to agree with reasons behind it provided. Business Development have a team coffee break once per week so could be discussed then.	Chief Exec to send email<br>Researcher to write email templates (share with those involved in organising package for comment)
**Seek out staff at their desk rather than email or phone – open door (linked to action above) (Intra & Org)**	"Walk, talk, email" slogan<br>Put out on stop-press, encourage staff to set location, so can easily check where a colleague is before going to see them	Day 1, week 1 – to continue for 12 weeks	Support and encouragement, setting an example	Hot-desking – locating staff might be a wasted trip<br>Staff expecting a colleague to help them immediately if turn up – might not be possible<br>Need for audit trail	Staff encouraged to set location so colleagues know if in office<br>Be mindful to check with colleagues that have time to talk<br>If need record of discussion, ok to put together a brief email highlighting key points	Email promoting this to come from Chief Exec (as above)
**Hold team meetings stood up or at least incorporate**	All team leaders to inform staff of this new initiative<br>If meetings are short e.g. less than 15 minutes – to	Day 1, week 1 – to continue for 12 weeks	Management support, team leaders to initiate	Some staff may not be able to stand for prolonged periods	Do not enforce, just make it optional, but encourage everyone	Team leaders to initiate<br>Directive to come from Chief Exec /

**some standing time into meetings (Org)**	have the whole meeting standing, if longer, ensure that some standing or moving time is incorporated into the agenda. Encourage walking 1:1s			Difficult to take notes during meetings when stood-up	that can to stand for short periods at least<br>If need to take some notes, sit for short periods	senior management
**Personal targets for steps/day and track activity using FitBit / pedometers and record steps each day (Intra & Inter)**	Individuals to set their own personal targets for steps per day and share with team / colleagues (maybe write on a white board, or a sheet pinned to the wall) – emphasis on personal achievements rather than competing with each other	Day 1, week 2 & every other week after that	Management support and encouragement required and also support from team leader	Not everyone may want to participate	Could involve those who are less interested in tracking steps by get them to stand and update the board / sheet with everyone's steps	Teams to initiate this
**Celebrate changes / achievements – tell us your sit less stories (Inter & Org)**	Personal stories of things you / your team are doing on Yammer or SYnet – could set-up a new group that encourages and celebrates successes<br>Include a hashtag - #sitlessSYHA	Day 1, week 3 & every other week after that	Would be helpful for manager / leadership to share their stories – gives permission / buy-in	Not everyone looks at Yammer or SYnet<br>Time	Talking about it in team meetings and agreeing who can share what<br>Group activities booking in advance in staff's diaries<br>Friendly competitions between teams / colleagues	All share stories and coordinate own Yammer posts.<br>Marketing/ Comms team to promote – DP to liaise
**Lunchtime walking or running group (Inter)**	Already a running group set-up – go 1 x week<br>Could set-up a weekly walking group – would	Day 1, week 4 – to continue for 12 weeks	Management support and encouragement	Only one shower and lack of changing facilities may put staff off the running group	If put off by lack of changing facilities – could signpost those	Need organisers of walking group to be set-up

	need to plan some walking routes – do certain things on the walks e.g. look for wildlife<br>These could all be advertised using Yammer or via news stories on the intranet				staff to the walking group instead	An SYHA running group has recently been set up by AP so link in with her
**Clear office / desk (Intra, Org, Env)**	Not keeping personal supply of stationary – have a team supply or use the stationary cupboard	Day 1, week 6	Managers to implement and buy-in	Staff too “attached” to their own stationary<br>Nowhere for a central stationary point to be put<br>No one takes responsibility for central point so staff don’t use and revert back to own supply	Find a place where stationary is easily accessed for all the team<br>Encourage staff to take responsibility for replacing items	Team leaders to initiate this<br>Need some directive from Chief Exec
**Changes in policies and guidelines (Org)**	Include something into the core brief about SYHA’s awareness that prolonged sitting is bad for your health and the importance of taking regular breaks and a summary of the initiatives that have been put in place (i.e. this document) – could make it a working document which evolves over time	Throughout 12-week period	Management would need to approve this			DP to liaise with Tim Gallimore to discuss this with as he looks at behaviours and culture change.

Note: the levels of influence each action related to is denoted as follows: "Intra" is intrapersonal, "Inter" is interpersonal, "Org" is organisational, and "Env" is environmental.
