# Supplementary material for: Understanding the Implementation of “Sit Less at Work” Interventions in Three Organisations: A Mixed Methods Process Evaluation
Source: Int J Environ Res Public Health. 2021 Jul 9;18(14):7361. doi: 10.3390/ijerph18147361 (PMC8304152; doi:10.3390/ijerph18147361)
Supplement: Supplementary file 1 [file ijerph-18-07361-s001.zip › Eval Paper_Table S3.pdf]

Table S3a. Local authority 12-week “Sit Less at Work” intervention summary

Actions to be initiated at the start and to continue throughout the 12-weeks:

- *Hold any team meetings over this 12-week period stood-up or incorporate standing/moving into these meetings (Org)*
- *Include improving health and wellbeing in 1:1s (Org)*
- *Include sitting less / moving more into workplace guidelines (Org)*

Week	Action 1	Action 2	Action 3	Action 4
**1**	Encourage staff to sit less / move more using Chief Exec’s weekly blog<br>(Intra, Org & Env)	Team step competition – new competition begins<br>(Inter)		
**2**	Encourage staff to sit less / move more using Chief Exec’s weekly blog	Team step competition – on-going		
**3**	Encourage staff to sit less / move more using Chief Exec’s weekly blog	Team step competition – on-going	Team standing breaks – whole team stands for 2-5 mins every hour<br>(Inter)	
**4**	Encourage staff to sit less / move more using Chief Exec’s weekly blog	Team step competition – winner selected	Team standing breaks – whole team stands for 2-5 mins every hour	Message over tannoy to remind everyone to stand / move (Tuesday at 11.30am)<br>(Org)

**5**	Encourage staff to sit less / move more using Chief Exec's weekly blog	Team step competition – new competition begins	Team standing breaks – whole team stands for 2-5 mins every hour	Message over tannoy to remind everyone to stand / move (Tuesday at 11.30am)
**6**	Encourage staff to sit less / move more using Chief Exec's weekly blog	Team step competition – on-going	Team standing breaks – whole team stands for 2-5 mins every hour	Message over tannoy to remind everyone to stand / move (Tuesday at 11.30am)
**7**	Encourage staff to sit less / move more using Chief Exec's weekly blog	Team step competition – on-going	Team standing breaks – whole team stands for 2-5 mins every hour	Message over tannoy to remind everyone to stand / move (Tuesday at 11.30am)
**8**	Encourage staff to sit less / move more using Chief Exec's weekly blog	Team step competition – winner selected	Team standing breaks – whole team stands for 2-5 mins every hour	Message over tannoy to remind everyone to stand / move (Tuesday at 11.30am)
**9**	Encourage staff to sit less / move more using Chief Exec's weekly blog	Team step competition – new competition begins	Team standing breaks – whole team stands for 2-5 mins every hour	Message over tannoy to remind everyone to stand / move (Tuesday at 11.30am)
**10**	Encourage staff to sit less / move more using Chief Exec's weekly blog	Team step competition – on-going	Team standing breaks – whole team stands for 2-5 mins every hour	Message over tannoy to remind everyone to stand / move (Tuesday at 11.30am)
**11**	Encourage staff to sit less / move more using Chief Exec's weekly blog	Team step competition – on-going	Team standing breaks – whole team stands for 2-5 mins every hour	Message over tannoy to remind everyone to stand / move (Tuesday at 11.30am)
**12**	Encourage staff to sit less / move more using Chief Exec's weekly blog	Team step competition – winner selected	Team standing breaks – whole team stands for 2-5 mins every hour	Message over tannoy to remind everyone to stand / move (Tuesday at 11.30am)

Table S3b. Local authority detailed action plan

Action	Content	Timing	Management involvement	Barriers	How to overcome barriers	Who to action
**Encourage staff to sit less / move more using a variety of different forms of comms (Intra, Org, Env)**	Group together a range of sit less/move more ideas which will be communicated with staff via: email from Chief Exec in her weekly blog; provide a link to webpage (via comms team); further email prompts from Chief Exec.<br>Sit less/move more ideas to be split into those that can be done in break/own times and those that can be done during non-break times. Break/own time ideas include: taking part in exercise classes / Pilates, active travel to work, lunchtime walk, play table tennis in the atrium, participate in walking/running group (already exists), “informal office sports” e.g. tea bag darts. Non-break time ideas include: talk not email, book meeting rooms on a floor you don’t work on, stairs instead of lift, download app to remind you to get up every hour (need to recommend an app), walk to any local meetings, use standing desks for work or meetings (need to highlight where these are), drink more water, stretches (need to recommend stretches).	Day 1, week 1 and weekly after that	Chief Exec will need to approve these communications	Concerns that middle management may not support this.<br>Those on remote sites across the borough may be excluded.<br>Emails that are telling you what to do may put some people off.	To be discussed in team meetings to highlight what is happening and why<br>Messages to be sent from Directors of each Directorate – to be reviewed at Directors meeting and an email to be sent addressing middle managers.<br>Extra effort will be required to communicate message to those on remote sites across the borough.<br>Ensure the emails are worded to make it clear these were ideas that came from a group of colleagues – so	Researcher to draft email template to be sent<br>Researcher to contact Chief Exec and Comms team to discuss how to disseminate information (flag up issue of including those on remote sites)<br>Researcher to liaise with Occupational Health / physio re. stretches

	Also to be included in communications – awareness raising about the health benefits of sitting less and moving more e.g. stress relief.<br>A link to these messages to remain at the end of the weekly blog for the entire 12-weeks.				staff know it is bottom-up rather than top-down.	
**Team step competition (Inter)**	To be undertaken within teams – but focus more on individual improvements and rather than who can do the most, so you're competing with yourself rather than each other.<br>Use iPhones, FitBits, pedometers (if available) to monitor steps.<br>Winner every 4 weeks – biggest improver week-on-week (3 x during 12-week period)<br>Will need champions in each team to ensure this happens	Day 1, week 1 – to continue for 12 weeks	Approval to run this will be required<br>Need to set-up as teams – would require a simple spreadsheet	Not everyone will have the available technology (iPhone, FitBit)	Check with Public Health to see if they have some leftover pedometers from a previous challenge.	Individual teams – would need champions in each team to action this
**Hold team meetings stood up or incorporate some standing time into meetings (Org)**	All team leaders to inform staff of this new initiative<br>If meetings are short e.g. less than 15 minutes – to have the whole meeting standing, if longer to ensure that some standing or moving time is incorporated into the agenda	Day 1, week 1 – to continue for 12 weeks	Directors support, team leaders to initiate	Some people may not be able to stand for prolonged periods	Do not enforce, just make it optional, but encourage everyone that can to stand for short periods at least	Directors disseminate at Full Area Manager meetings, then message relayed to team leaders in local management meetings

**Include improving health and wellbeing in 1:1s (Org)**	To have as standard some question relating to how to improve health and wellbeing at work with particular reference to sitting less and moving more.	Day 1, week 1 – to continue for the 12 weeks	Approval from Chief Exec and HR	Managers may need training or will at least need to be made aware of this	HR to provide advice on information managers would need to successfully complete this in the 1:1	Researcher to liaise with Chief Exec and HR (who create the basic framework)
**Include sitting less / moving more into workplace guidelines (Org)**	Include information relating to sitting less / moving more at work in local workplace health and wellbeing guidance, acknowledging that local authority is aware that prolonged sitting is bad for health and therefore the importance of taking regular breaks and a summary of the initiatives that have been put in place (i.e. this document)	Day 1, week 1 – to continue for the 12 weeks	Approval from Chief Exec, HR and/or Occupational Health	Would need HR and/or Occupational Health approval and involvement – may not have time/be a priority	Need to liaise with HR and Occupational Health to discuss	Researcher to liaise with HR, Occupational Health and Chief Exec
**Team standing breaks (Inter)**	Every hour, whole team stands for 2-5 mins and stretches (need to provide recommended stretches). Will need to have a champion / series of champions in each team to lead this and ensure it happens	Day 1, week 3 – to continue for 12 weeks	Approval to run this will be required	Health and Safety<br>Time / work pressures	Link in with occupational health and physio<br>Schedule into diaries, team leader to support this so all staff feel able to participate – need a champion in each team	Individual teams – would need champions in each team to action this<br>Researcher to liaise with Occupational Health / physio re. stretches

***Message over tanoy to remind everyone to stand / move (Org)**	Message over tanoy e.g. "Hi di hi" which is the prompt to remind everyone to stand-up and move. To occur every Tuesday at 11am.	Day 2, week 4, then weekly after that	Approval from Chief Exec	Some may not want to join in as too busy / no time	That's ok, it should be optional	Researcher to liaise with Facilities Management and Chief Exec

**This idea was deemed unfeasible by the implementation team so was removed from the final action plan*

Note: the levels of influence each action related to is denoted as follows: "Intra" is intrapersonal, "Inter" is interpersonal, "Org" is organisational, and "Env" is environmental.
