# Supplementary material for: Understanding the Implementation of “Sit Less at Work” Interventions in Three Organisations: A Mixed Methods Process Evaluation
Source: Int J Environ Res Public Health. 2021 Jul 9;18(14):7361. doi: 10.3390/ijerph18147361 (PMC8304152; doi:10.3390/ijerph18147361)
Supplement: Supplementary file 1 [file ijerph-18-07361-s001.zip › Eval Paper_Table S4.pdf]

Table S4. Participation numbers during the intervention and reasons for dropouts by organisation

Organisation	T0 participants (n)	T1 participants (n)	T2 participants (n)
**Small business**	5 (data available for n=5)	5 (data available for n=4; 1 activPAL3™device did not work)	4 (data available for n=2; 2 activPAL3™ devices did not work; 1 person left organisation between T1 and T2)
**Charity**	11 (data available for n=10; 1 person did not have time to complete data collection and dropped out of the study)	10 (data available for n=8; 1 person left organisation and did not return activPAL3™device, 1 person did not have time to complete data collection; 1 person dropped between T0 and T1 without providing a reason)	8 (data available for n=8; 2 people dropped out between T1 and T2 without providing a reason)
**Local authority**	41 (data available for n=37; 2 activPAL3™ devices did not work, 1 person left the organisation and did not return device, 1 person dropped out due to irritation from the dressing used to adhere device to skin)	38 (data available for n=28; 3 people were on annual leave, 3 people opted out, 2 people had no time, 1 activPAL3™ device did not work, 1 device was not returned; 3 people dropped out between T0 and T1 without providing a reason)	28 (data available for n=21; 5 people opted out, 1 person left the organisation, 1 activPAL3™ device did not work; 10 people dropped out between T1 and T2 without providing a reason)

Note: Number of participants where data were available is determined by the receipt of valid activPAL3™ data
