# Supplementary material for: Understanding the Implementation of “Sit Less at Work” Interventions in Three Organisations: A Mixed Methods Process Evaluation
Source: Int J Environ Res Public Health. 2021 Jul 9;18(14):7361. doi: 10.3390/ijerph18147361 (PMC8304152; doi:10.3390/ijerph18147361)
Supplement: Supplementary file 1 [file ijerph-18-07361-s001.zip › Eval Paper_Table S5.pdf]

Table S5a. Awareness of the "Sit Less at Work" intervention in the small business (total responses n=5)

**Awareness of:**	**Yes (n)**
The "Sit Less at Work" intervention as a whole	5
Regular emails from Managing Director	3
Using computer prompts	2
Using exercise ball instead of chair	2
Competitions e.g., computer games, ping pong, press-ups	4
Rota for walking to the shops for lunch/snacks	2
Exercises with office dumbbells	5
Using wireless headsets	2
Social media promotion of "Sit Less at Work"	4
Inclusion of "Sit Less at Work" as an agenda item in team meetings and in the Wellbeing Policy	2
Other initiative not listed above	2
Unaware of any "Sit Less at Work" initiatives	0

Table S5b. Awareness of the "Sit Less at Work" intervention in the charity (total responses n=9)

**Awareness of:**	**Yes (n)**
The "Sit Less at Work" intervention as a whole	9
Regular communications re. "Sit Less at Work" intervention	8
Encouragement to set personal targets for steps per day	3
Sit less success stories being celebrated on the intranet	1
Encouragement to join or set-up lunchtime walking/running group	3
The clear office / desk policy	5
Team meetings incorporated periods of standing/moving	4
Policy changes to support sitting less at work	1
Other initiative not listed above	0

Unaware of any “Sit Less at Work” initiatives	0

Table S5c. Awareness of the “Sit Less at Work” intervention in the local authority (total responses n=28)

**Awareness of:**	**Yes (n)**
The “Sit Less at Work” intervention as a whole	25
Regular communications with suggestions to sit less at work	12
Encouragement to lead or participate in step competitions	2
Team standing breaks	2
Posters near photocopiers to encourage stretches	13
Posters and stands in meeting rooms to encourage sitting less and moving more	10
The inclusion of some standing or moving in team meetings	2
The inclusion of sitting less and moving more in 1:1s	1
The inclusion of sitting less and moving more in workplace guidelines	5
Other initiative not listed above	6
Unaware of any “Sit Less at Work” initiatives	2
